# Differential Pattern of Cell Death and ROS Production in Human Airway Epithelial Cells Exposed to Quinones Combined with Heated-PM2.5 and/or Asian Sand Dust

**DOI:** 10.3390/ijms241310544

**Published:** 2023-06-23

**Authors:** Akiko Honda, Ken-ichiro Inoue, Makoto Higashihara, Takamichi Ichinose, Kayo Ueda, Hirohisa Takano

**Affiliations:** 1 Graduate School of Engineering, Kyoto University, Kyoto 615-8540, Japan; 2 School of Nursing, University of Shizuoka, Shizuoka 422-8526, Japan; 3 Graduate School of Global Environmental Studies, Kyoto University, Kyoto 615-8540, Japan; 4 Department of Health Science, Oita University of Nursing and Health Sciences, Oita 870-1201, Japan; 5 Department of Hygiene, Graduate School of Medicine, Hokkaido University, Hokkaido 060-8638, Japan; 6 Institute for International Academic Research, Kyoto University of Advanced Science, Kyoto 615-8577, Japan

**Keywords:** quinones, h-PM2.5, h-ASD, inflammation, apoptosis, reactive oxidative stress

## Abstract

The combined toxicological effects of airborne particulate matter (PM), such as PM2.5, and Asian sand dust (ASD), with surrounding chemicals, particularly quinones, on human airway epithelial cells remain underexplored. In this study, we established an in vitro combination exposure model using 1,2-naphthoquinones (NQ) and 9,10-phenanthroquinones (PQ) along with heated PM (h-PM2.5 and h-ASD) to investigate their potential synergistic effects. The impacts of quinones and heated PM on tetrazolium dye (WST-1) reduction, cell death, and cytokine and reactive oxygen species (ROS) production were examined. Results revealed that exposure to 9,10-PQ with h-PM2.5 and/or h-ASD dose-dependently increased WST-1 reduction at 1 μM compared to the corresponding control while markedly decreasing it at 10 μM. Higher early apoptotic, late apoptotic, or necrotic cell numbers were detected in 9,10-PQ + h-PM2.5 exposure than in 9,10-PQ + h-ASD or 9,10-PQ + h-PM2.5 + h-ASD. Additionally, 1,2-NQ + h-PM2.5 exposure also resulted in an increase in cell death compared to 1,2-NQ + h-ASD and 1,2-NQ + h-PM2.5 + h-ASD. Quinones with or without h-PM2.5, h-ASD, or h-PM2.5 + h-ASD significantly increased ROS production, especially with h-PM2.5. Our findings suggest that quinones, at relatively low concentrations, induce cell death synergistically in the presence of h-PM2.5 rather than h-ASD and h-PM2.5 + h-ASD, partially through the induction of apoptosis with increased ROS generation.

## 1. Introduction

Particulate matter (PM) induces adverse health effects, including respiratory toxicity. PM is a complex mixture of carbon particles in which various organic compounds, including polycyclic aromatic hydrocarbons (PAHs), are adsorbed. PM2.5 is a complex mixture of solid and liquid airborne particles, and different PM2.5 components have different effects on cell inflammatory responses [1]. In China, Xi’an is one of the most heavily polluted cities and has experienced poor air quality in recent years [2]. Several studies in Xi’an have examined the negative effects of ambient air pollutants on various outcomes, including mortality and respiratory diseases [3,4,5]. However, few studies have focused on specific hazards associated with certain PM2.5 components and their influence on inflammatory processes and oxidative stress [6]. In the atmosphere, PAHs are converted to nitro-PAHs, which are more mutagenic and carcinogenic than PAHs because they contain nitrogen molecules. PAHs in the air can also be converted to their respective quinone forms through photooxidation. Additionally, PAHs are metabolized in vivo via transdihydrodiols by cytochrome P450 (CYP) and epoxide hydrolases and are finally converted to PAH quinones by two-electron reductases such as aldo-keto reductase [7].

Quinones are by-products of the partial combustion of biomass and fossil fuels, petroleum products (especially diesel), PAHs, and polychlorinated biphenyls. These quinones are ubiquitous environmental pollutants, and humans are increasingly getting exposed to them, posing health risks, including cancer. Toxicological studies of environmentally occurring quinones, such as naphthoquinones (1,2-NQ and 1,4-NQ) and phenanthroquinones (9,10-PQ), have reported that these compounds can cause mutations, DNA damage, chromosomal aberrations, and altered signal transduction pathways. These toxic effects have been attributed to their electrophilic and redox-cycling properties and have been linked to various cancers, including leukemias [8,9,10,11]. Previously, we examined the respiratory effects of several quinone compounds and found that transairway exposure to certain quinone compounds can induce airway inflammation [12] and exacerbate allergic airway inflammation [12,13,14]. On the other hand, few studies have investigated the combined toxicological effects of particles and chemicals. In other words, the hazardous effects of airborne chemicals should be estimated/compared in combination with suspended types of PM, considering their main form of existence in the environment. With the above background, this study aimed to examine the combined respiratory effects of quinones (1,2-NQ and 9,10-PQ) and PM using a combined in vitro exposure model. PM2.5 and Asian sand dust (ASD) were heated to exclude chemicals such as PAHs. We assessed the respiratory effects of this combination based on changes in tetrazolium dye (WST-1) as an external electron acceptor for reduction, cell death patterns, inflammation, and oxidative stress in vitro.

## 2. Results

### 2.1. The Combined Effect of Quinone and h-PM2.5 and/or h-ASD on the WST-1 Reduction Assay of Bronchial Epithelial Cells

BEAS-2B cells were treated with varying concentrations of 9,10-PQ alone, 9,10-PQ + h-PM2.5, and/or h-ASD, and their WST-1 reduction was studied. 9,10-PQ with or without h-PM2.5, h-ASD, or h-PM2.5 + h-ASD dose-dependently increased WST-1 reduction vs. corresponding controls (*p* < 0.01: 9,10-PQ at 0.1 and 1 μM; 9,10-PQ + h-PM2.5 at 0.1 and 1 μM; 9,10-PQ + h-ASD at 1 μM; and 9,10-PQ + h-PM2.5 + h-ASD at 0.1 and 1 μM). However, the WST-1 reduction was markedly decreased at 10 μM (*p* < 0.01) when compared to the corresponding control for all groups.

**Figure 1:**
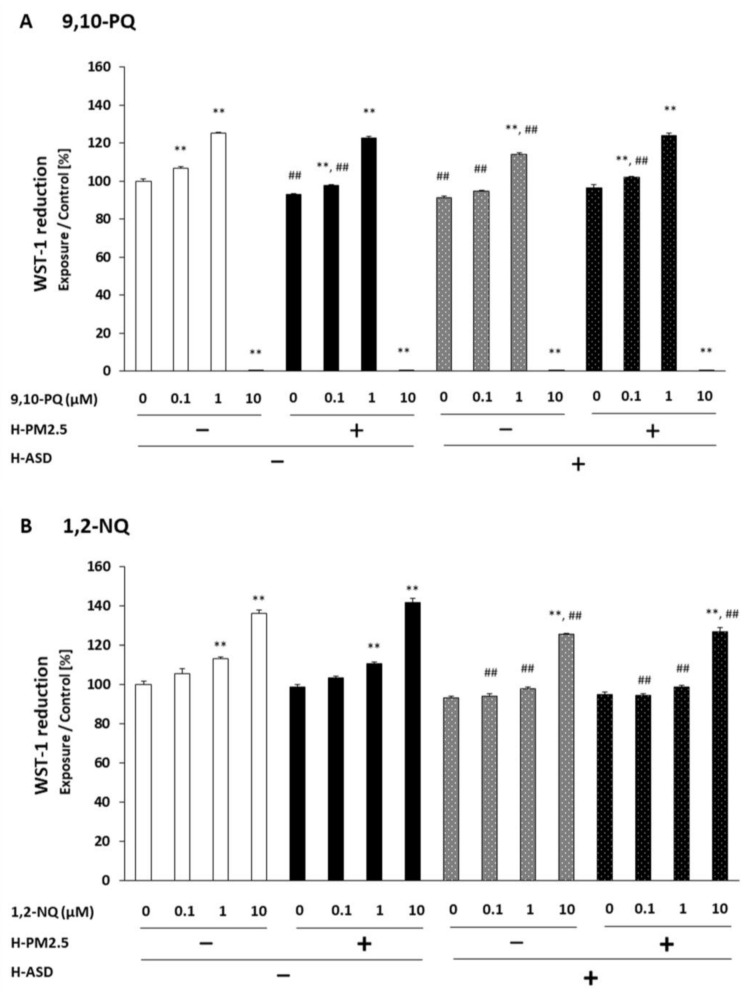
The combined effect of quinone and h-PM2.5 and/or h-ASD on the WST-1 reduction of bronchial epithelial cells. Cells were treated with the specified concentrations of quinone, h-PM2.5, and/or h-ASD for 24 h, and their WST-1 reduction was assessed. (**A**) 9,10-PQ; (**B**) 1,2-NQ. The data are represented as the percentage of the WST-1 reduction of the control (0 μM 9,10-PQ or 1, 2-NQ, and 0 μg/mL h-PM2.5 and/or h-ASD) and shown as the mean ± standard error of the mean (SEM) from 4 individual cultures. ** *p* < 0.01 vs. corresponding control; ## *p* < 0.01 vs. quinone at the same concentration. Asian dust sand (ASD); particulate matter (PM); naphthoquinones (NQ); phenanthroquinones (PQ); tetrazolium dye (WST).

When comparing h-PM2.5, h-ASD, and h-PM2.5 + h-ASD in the absence of 9,10-PQ, a lower WST-1 reduction was observed after h-PM2.5 or h-ASD exposure than after vehicle exposure (*p* < 0.01). At 0.1 µM, a lower WST-1 reduction was seen after 9,10-PQ + h-PM2.5 and/or + h-ASD exposure than after 9,10-PQ exposure (*p* < 0.01). At 1 µM, a lower WST-1 reduction was observed after 9,10-PQ + h-ASD exposure than after 9,10-PQ exposure (*p* < 0.01) (Figure 1A).

We treated BEAS-2B cells with varying concentrations of 1,2-NQ alone, 1,2-NQ + h-PM2.5, and/or h-ASD and studied WST-1 reduction. 1,2-NQ with or without h-PM2.5, h-ASD, or h-PM2.5 + h-ASD dose-dependently increased WST-1 reduction vs. corresponding controls (*p* < 0.01 for 1,2-NQ at 1 and 10 μM, 1,2-NQ + h-PM2.5 at 1 and 10 μM, 1,2-NQ + h-ASD at 10 μM, and 1,2-NQ + h-PM2.5 + h-ASD at 10 μM).

A relative comparison between compounds showed that at 0.1, 1, and 10 µM, lower WST-1 reduction was observed after exposure to 1,2-NQ + h-ASD and 1,2-NQ + h-PM2.5 + h-ASD than after exposure to 1,2-NQ alone (*p* < 0.01) (Figure 1B).

### 2.2. The Combined Effect of Quinone and h-PM2.5 and/or h-ASD on Apoptosis and Necrosis of Airway Epithelial Cells

**Figure 2:**
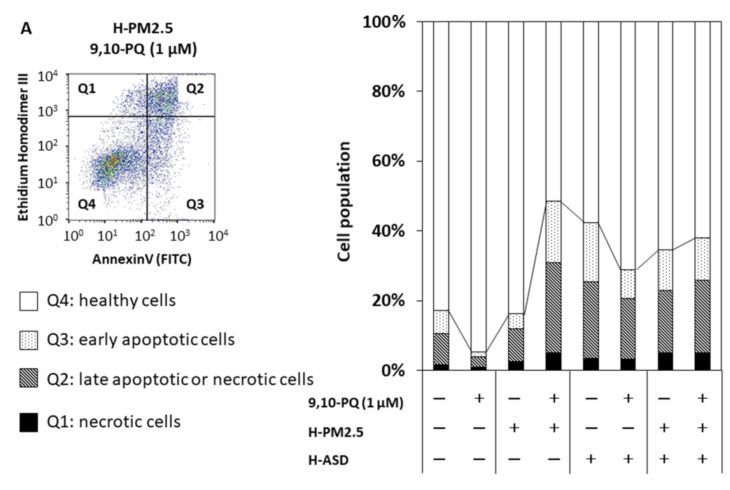

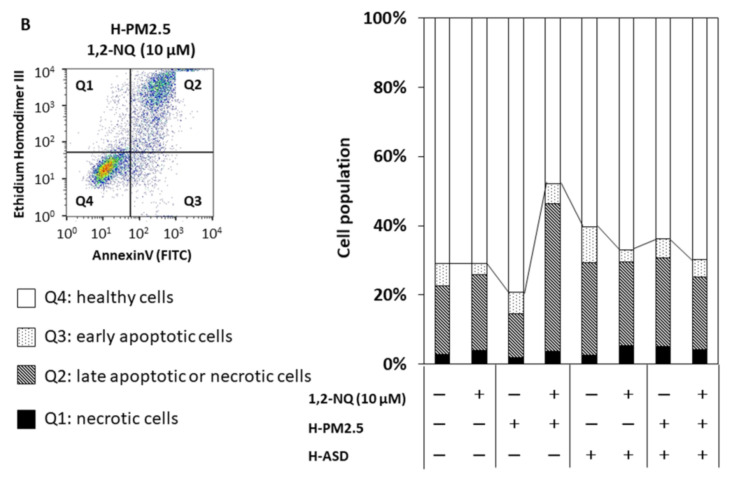
The combined effect of quinone and h-PM2.5 and/or h-ASD on apoptosis and necrosis of airway epithelial cells at 24 h after exposure. (**A**) 9,10-PQ, (**B**) 1,2-NQ. Dot-plot of flow cytometry and cell population: Q1, necrotic cells; Q2, late apoptotic or necrotic cells; Q3, early apoptotic cells; Q4, healthy cells.

At 1 µM of 9,10-PQ, 9,10-PQ + h-PM2.5 increased the number of early apoptotic cells, late apoptotic or necrotic cells, and necrotic cells compared with h-PM2.5, whereas the numbers of early apoptotic cells and late apoptotic or necrotic cells after exposure to 9,10-PQ, 9,10-PQ + h-ASD, were lower than those after vehicle and h-ASD, respectively (Figure 2A). Exposure to h-ASD or h-PM2.5 + h-ASD increased the number of late apoptotic, or necrotic cells vs. vehicle, whereas the number after exposure to h-PM2.5 was similar to that after vehicle. Notably, 9,10-PQ + h-PM2.5 increased the number of early apoptotic cells, late apoptotic, or necrotic cells among 9,10-PQ, 9,10-PQ + h-ASD, and 9,10-PQ + h-PM2.5 + h-ASD. At 10 μM of 9,10-PQ, exposure to 9,10-PQ, 9,10-PQ + h-PM2.5, 9,10-PQ + h-ASD, and 9,10-PQ + h-PM2.5 + h-ASD all increased the numbers compared with exposures to the corresponding control (Supplementary Figure S2).

At 10 µM of 1,2-NQ, 1,2-NQ + h-PM2.5 increased the number of late apoptotic or necrotic cells, and necrotic cells compared with h-PM2.5, whereas the numbers of early apoptotic cells or late apoptotic or necrotic cells after exposure to 1,2-NQ + h-ASD and 1,2-NO + h-PM2.5 + h-ASD were lower than those after h-ASD and h-PM2.5 + h-ASD, respectively (Figure 2B). Exposure to h-ASD or h-PM2.5 + h-ASD increased the number of late apoptotic or necrotic cells vs. vehicle, whereas the number after exposure to h-PM2.5 decreased after vehicle. Notably, 1,2-NQ + h-PM2.5 increased the number of late apoptotic or necrotic cells among 1,2-NQ, 1,2-NQ + h-ASD, 1,2-NQ + h-PM2.5 + h-ASD.

### 2.3. The Combined Effect of 9,10-PQ and h-PM2.5 and/or h-ASD on Cytokine Production

**Figure 3:**
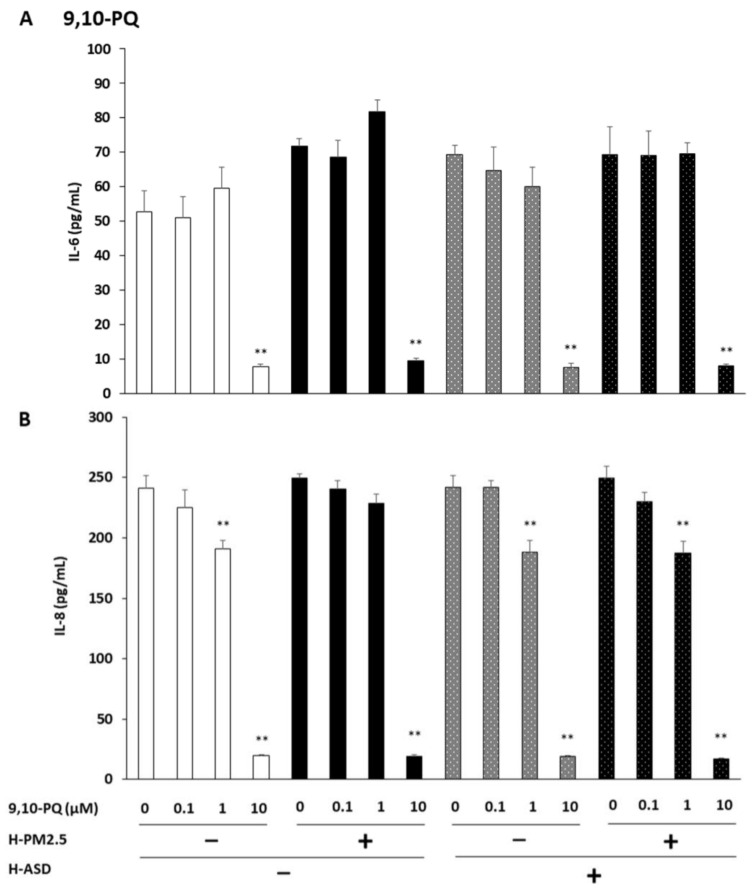
The combined effect of 9,10-PQ and h-PM2.5 and/or h-ASD on interleukin (IL)-6 and IL-8 production from human bronchial epithelial cells. The protein levels of (**A**) IL-6 and (**B**) IL-8 in the culture supernatants after exposure to 9,10-PQ and h-PM2.5 and/or h-ASD for 24 h were measured using an enzyme-linked immunosorbent assay (ELISA). The data are expressed as the mean ± SEM of 4 individual cultures. ** *p* < 0.01 vs. the corresponding control.

We evaluated IL-6 production from the cells after exposure to 0.1, 1, and 10 µM of 9,10-PQ + h-PM2.5 and/or h-ASD. We observed no significant difference after exposure to 0.1 and 1 μM of all compounds compared to the corresponding control. At 10 µM, IL-6 production decreased after exposure to all compounds compared to the corresponding control (*p* < 0.01). A relative comparison between compounds showed that at 0.1, 1, and 10 µM, the production was comparable between 9,10-PQ and combined exposure at every dose (Figure 3A).

IL-8 production was examined after treatment with 0.1, 1, and 10 µM of 9,10-PQ + h-PM2.5 and/or h-ASD. No significant difference was observed after exposure to 0.1 µM of all compounds compared to the corresponding control. At 1 µM of 9,10-PQ, the IL-8 levels decreased in the 9,10-PQ, 9,10-PQ + h-ASD, and 9,10-PQ + h-PM2.5 + ASD-treated cells (*p* < 0.01) compared to the corresponding control. At 10 µM of 9,10-PQ, the IL-8 levels decreased in the 9,10-PQ, 9,10-PQ + h-PM2.5, 9,10-PQ + h-ASD, and 9,10-PQ + h-PM2.5 + h-ASD-treated cells (*p* < 0.01) vs. the corresponding control. Relative comparisons between compounds showed that at 0.1, 1, and 10 µM, the production was comparable between 9,10-PQ and the combined exposure at every dose (Figure 3B).

### 2.4. The Combined Effect of 1,2-NQ and h-PM2.5 and/or h-ASD on Cytokine Production

**Figure 4:**
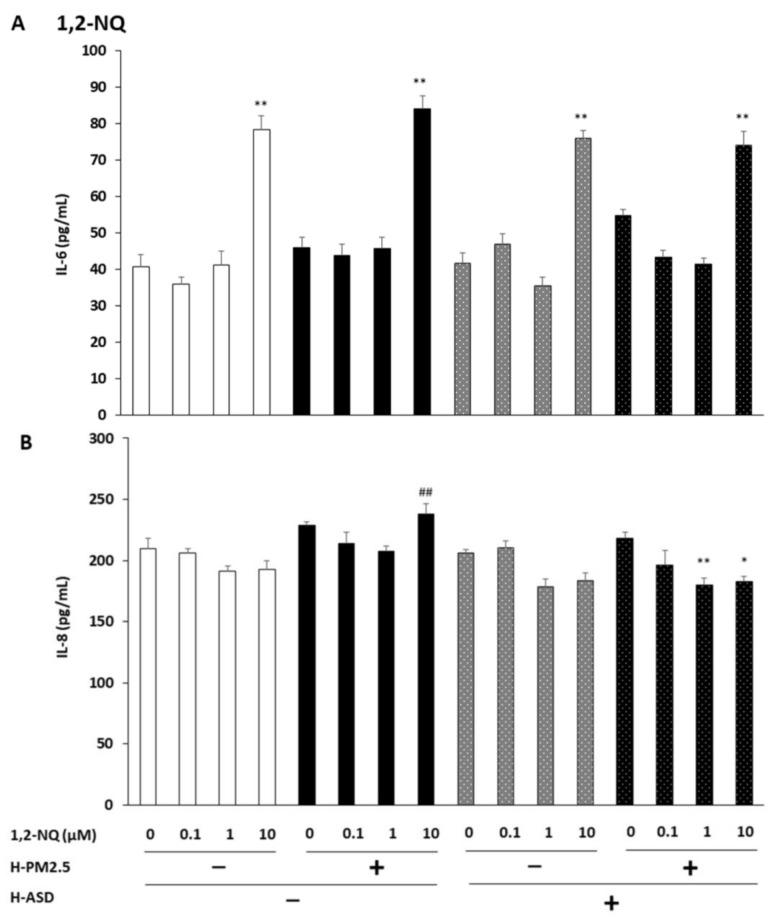
The combined effect of 1,2-NQ and h-PM2.5 and/or h-ASD on the levels of IL-6 and IL-8 production from human bronchial epithelial cells. The protein levels of (**A**) IL-6 and (**B**) IL-8 in the culture supernatants after exposure to 1,2-NQ and h-PM2.5 and/or h-ASD for 24 h were measured using ELISA. The data are expressed as the mean ± SEM of four individual cultures. * *p* < 0.05; ** *p* < 0.01 vs. corresponding control; ## *p* < 0.01 vs. quinone at the same concentration.

We evaluated the production of IL-6 from cells exposed to 0.1, 1, and 10 µM of 1,2-NQ + h-PM2.5 and/or h-ASD. We observed no significant difference in IL-6 production after exposure to 0.1 and 1 μM of all compounds compared to the corresponding control. However, at 10 µM of 1,2-NQ, IL-6 production increased significantly compared to the corresponding control after exposure to all compounds (*p* < 0.01). Relative comparison between compounds showed that at 0.1, 1, and 10 µM of 1,2-NQ, the production was similar between 1,2-NQ and the combined exposure at every dose (Figure 4A).

IL-8 production was examined after treatment with 0.1, 1, and 10 µM of 1,2-NQ, 1,2-NQ + h-PM2.5, and/or h-ASD. No significant difference was found after exposure to 0.1 µM of all compounds compared to the corresponding control. At 1 and 10 µM of 1,2-NQ, IL-8 levels were decreased in 1,2-NQ + h-PM2.5 + h-ASD-treated cells (*p* < 0.01 at 1 µM; *p* < 0.05 at 10 µM). Relative comparison between compounds showed that at 10 µM of 1,2-NQ, the production was higher following 1,2-NQ + h-PM2.5 exposure than following 1,2-NQ exposure (Figure 4B). 

### 2.5. The Combined Effect of 9,10-PQ and h-PM2.5 and/or h-ASD on ROS Production

**Figure 5:**
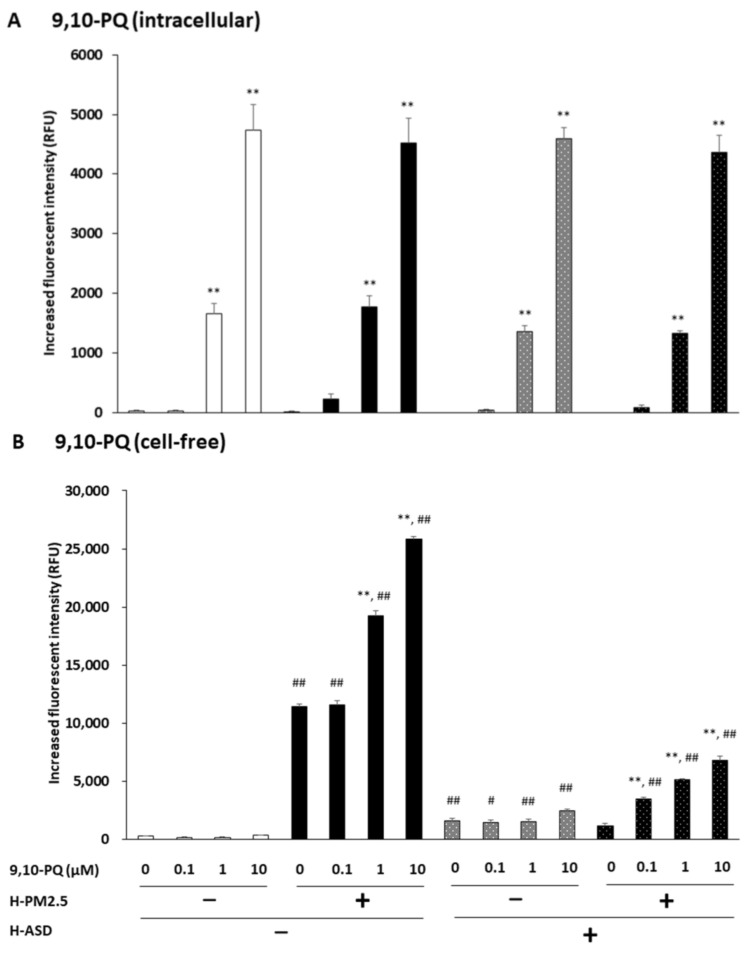
The combined effect of 9,10-PQ and h-PM2.5 and/or h-ASD on reactive oxygen species (ROS) production by airway epithelial cells. Intracellular (**A**) and cell-free (**B**) ROS production. The data are presented as the mean ± SEM from four individual cultures. ** *p* < 0.01 vs. corresponding control; # *p* < 0.05; ## *p* < 0.01 vs. quinone at the same concentration.

BEAS-2B cells were exposed to varying concentrations of 9,10-PQ + h-PM2.5 and/or h-ASD, and their ROS production was examined. Exposure to 9,10-PQ with or without h-PM2.5, h-ASD, or h-PM2.5 + h-ASD dose-dependently increased ROS production (*p* < 0.01; exposure to 1 and 10 μM of all compounds) compared to the corresponding control. However, no significant differences in ROS production were found between the groups treated with 9,10-PQ with or without h-PM2.5, h-ASD, or h-PM2.5 + h-ASD (Figure 5A).

In the cell-free condition, 9,10-PQ with h-PM2.5 or h-PM2.5 + h-ASD dose-dependently increased ROS production (*p* < 0.01) at concentrations of 1 and 10 μM of 9,10-PQ + h-PM2.5 and 0.1, 1, and 10 μM of 9,10-PQ + h-PM2.5 + h-ASD compared to the corresponding control (Figure 5B).

When comparing h-PM2.5, h-ASD, and h-PM2.5 + h-ASD in the absence of 9,10-PQ, higher ROS production was observed in h-PM2.5 or h-ASD than in vehicles (*p* < 0.01). At 0.1 μM, ROS levels increased in 9,10-PQ + h-PM2.5 (*p* < 0.01), 9,10-PQ + h-ASD (*p* < 0.05), or 9,10-PQ + h-PM2.5 + h-ASD (*p* < 0.01) compared with those in 9,10-PQ alone. At 1 μM and 10 μM, ROS levels increased in 9,10-PQ + h-PM2.5, 9,10-PQ + h-ASD, or 9,10-PQ + h-PM2.5 + h-ASD compared with those in 9,10-PQ alone (*p* < 0.01; Figure 5B). Furthermore, the increase in ROS levels was notable at 1 μM and 10 μM of 9,10-PQ + h-PM2.5 or 9,10-PQ + h-PM2.5 + h-ASD (*p* < 0.01 vs. 9,10-PQ alone).

### 2.6. The Combined Effect of 1,2-NQ and h-PM2.5 and/or h-ASD on ROS Production

**Figure 6:**
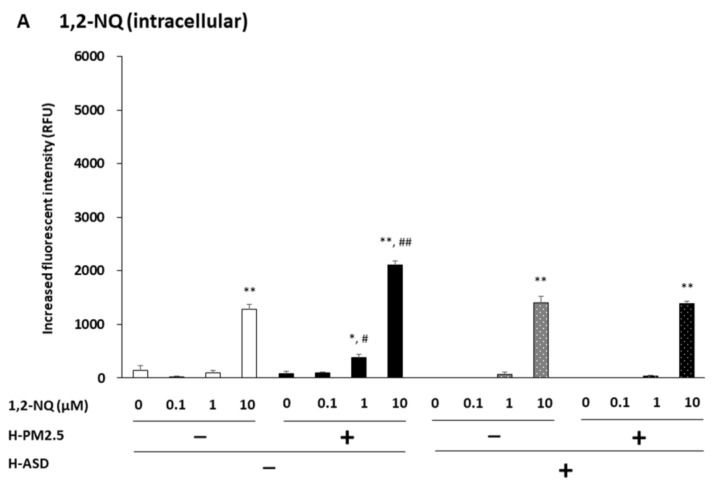

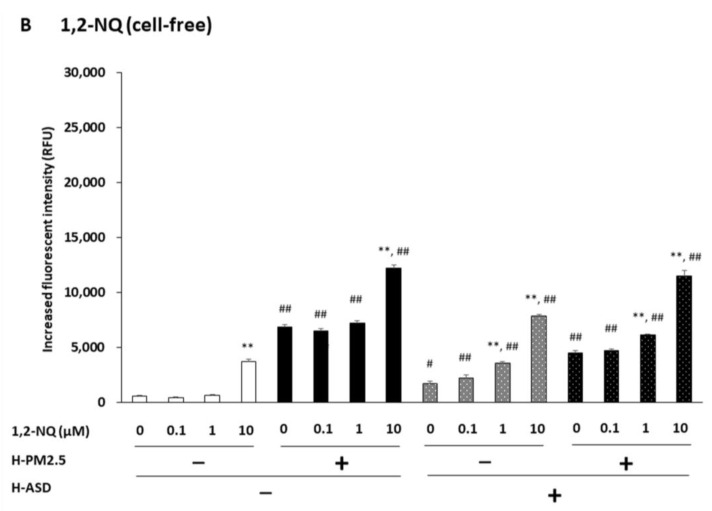
The combined effect of 1,2-NQ and h-PM2.5 and/or h-ASD on ROS production by airway epithelial cells. Intracellular (**A**) and cell-free (**B**) ROS production. The data are expressed as the mean ± SEM from four individual cultures. * *p* < 0.05; ** *p* < 0.01 vs. corresponding control; # *p* < 0.05; ## *p* < 0.01 vs. quinone at the same concentration.

BEAS-2B cells were treated with varying concentrations of 1,2-NQ + h-ASD, 1,2-NQ + h-PM2.5, and/or h-ASD, and ROS production was examined. Treatment with 1,2-NQ with or without h-PM2.5, h-ASD, or h-PM2.5 + h-ASD dose-dependently increased ROS production (*p* < 0.01; exposure to 10 μM in all compounds, *p* < 0.05; exposure to 1 μM in 1, 2-NQ + h-PM2.5) compared to the corresponding control. In the comparison at 1 and 10 µM of 1,2-NQ, ROS levels increased significantly after exposure to 1,2-NQ + h-PM2.5 compared to exposure to 1,2-NQ alone (Figure 6A).

In the cell-free condition, 1,2-NQ with or without PM2.5, h-ASD, or h-PM2.5 + h-ASD dose-dependently increased ROS production (*p* < 0.01). This effect was observed at 10 µM of 1,2-NQ, 10 µM of 1,2-NQ + h-PM2.5, and 1 and 10 µM of 1,2-NQ + h-ASD and 1,2-NQ + h-PM2.5 + h-ASD (*p*< 0.01) compared to the corresponding control (Figure 6B).

In the comparison among h-PM2.5, h-ASD, and h-PM2.5 + h-ASD in the absence of 1,2-NQ, higher ROS production was observed in h-PM2.5 (*p*< 0.01), h-ASD (*p* < 0.05), or h-PM2.5 + h-ASD (*p*< 0.05) than in vehicle. At 0.1 μM, ROS levels increased significantly in 1,2-NQ + h-PM2.5 (*p* < 0.01), 1,2-NQ + h-ASD (*p* < 0.01), or 1,2-NQ + h-PM2.5 + h-ASD (*p* < 0.01) compared to 1,2-NQ alone. At 10 μM, ROS levels increased significantly in 1,2-NQ + h-PM2.5, 1,2-NQ + h-ASD, or 1,2-NQ + h-PM2.5 + ASD compared to 1,2-NQ alone (*p* < 0.01; see Figure 6B).

## 3. Discussion

This study found that exposure to 9,10-PQ + h-PM2.5 and/or h-ASD dose-dependently increased WST-1 reduction at 1 μM compared to the corresponding control but markedly decreased it at 10 μM. At 1 μM, exposure to 9,10-PQ + h-PM2.5 increased the number of late apoptotic or necrotic cells compared among all groups. Exposure to 1,2-NQ + h-PM2.5 and/or h-ASD dose-dependently increased WST-1 reduction compared to the corresponding control. 1,2-NQ at 10 μM + h-PM2.5 increased the number of late apoptotic or necrotic cells compared among all groups. Quinones with or without h-PM2.5, h-ASD, or h-PM2.5 + h-ASD dose-dependently increased ROS production compared to the corresponding control. In the cell-free condition, quinones with or without h-PM2.5, h-ASD, or h-PM2.5 + h-ASD also dose-dependently increased ROS production compared to the corresponding control, with quinones + h-PM2.5 showing a significantly greater increase than quinones + h-ASD or h-PM2.5 + h-ASD. 

Although PMs, including PM2.5 and ASD, have respiratory toxicity, relatively few studies have investigated the responsible components, such as chemicals and heavy metals, in or around PMs contributing to this toxicity [15,16]. Other groups, including ours, have shown that the chemical components of PM2.5 have toxic potential and synergistically exacerbate allergic models in vivo [17,18,19,20]. However, most of these studies have examined the individual effects of these components. In the environment, particularly the atmosphere, these components commonly exist with suspended PMs such as PM2.5 and ASD and enter the body through the intestinal and respiratory routes, making it difficult to investigate their health effects in the real world. Therefore, studying the combined effects of these compounds and PMs can provide valuable and precise information about the mechanism of PM toxicity. To stimulate realistic effects, we preferentially selected two potential quinones and examined the combined effects of these quinones with heated PM2.5 and/or ASD in vitro. We used heated PMs in order to exclude some bioeffects from other materials/microorganisms attached to these PMs. In fact, we observed that the heating procedure of PM at 360 °C eliminated almost all PAHs, endotoxins, and fungus and also reduced toxicity [21]. As a result, this in vitro exposure model may be applied to other chemicals and/or metals, such as PAHs. In a study, IL-6 and IL-8 release from airway epithelial cells caused by organic extracts from PM2.5 were reduced by a metal chelator [22]. This finding indicates that a combination of organic components and metals in PM2.5 may lead to stronger proinflammatory responses. Indeed, we have previously shown that coexposure to cadmium (Cd) and 9,10-PQ additively/synergistically increased proinflammatory responses in airway epithelial cells, whereas coexposure to Cd and phenanthrene resulted in no acceleration applying to the same in vitro system as the current one [23]. Accordingly, the metal may be selected for the current model in the future. Furthermore, the combined exposure model can examine the effects of these compounds even at relatively low concentrations. Although our in vitro studies have shown that 1–10 μM of 9,10-PQ or 1,2-NQ induces a significant increase in the number of apoptotic or necrotic cells, these concentrations are lower than those reported by other groups [24,25].

In this study, exposure to 9,10-PQ and 1,2-NQ dose-dependently increased WST-1 reduction. However, exposure to both h-PM2.5 and h-ASD did not affect this increase. Tan and Berridge [26] reported that quinone can increase WST-1 reduction via NAD(P)H quinone oxidoreductase-1 (NQO-1) at optimum concentrations. Above these concentrations, inhibitory effects are observed. The present study also indicated that decreased WST-1 reduction correlates with increased cell death. It is possible that at lower concentrations, quinones disrupt the redox cycle while exhibiting strong toxicities at high concentrations. Furthermore, the results of IL-6 and IL-8 production or release from bronchial epithelial cells were similar to those of WST-1 reduction, showing an overall trend. This dose (50 μg/mL) of both particles may not influence the WST-1 reduction increased by quinones. Dose-dependent studies are necessary to determine the synergistic effects of these components. Additionally, in vivo studies using a pulmonary route of exposure may yield different results because the respiratory system has various resident cells, such as macrophages, leukocytes, lymphocytes, and endothelial cells.

Exposure to h-ASD or h-PM2.5 + h-ASD increased the number of late apoptotic or necrotic cells compared to vehicles. Conversely, in the cell-free condition, h-ASD increased ROS production compared to vehicle, although the value was considerably lower than that of h-PM2.5 at 50 μg/mL. Previous studies have demonstrated that h-ASD exposure induces apoptosis of epithelial cells, concomitant with increased ROS production [27,28]. However, Piao et al. [29] showed that exposure of human keratinocytes to PM2.5 increased intracellular ROS production, leading to endoplasmic reticulum stress, mitochondrial damage, autophagy, and cell apoptosis. In the present study, 50 μg/mL of h-ASD, but not h-PM2.5, increased apoptotic changes in epithelial cells. Although h-ASD at this dose can induce apoptosis or necrosis of epithelial cells, its effects on the cells may not be mediated by ROS hyperproduction. These results indicate that PMs can induce different types of cell deaths and injuries; thus, further studies are needed.

Interestingly, co-exposure to quinones and PM2.5 markedly induced late apoptosis or necrosis of cells. Previous studies have shown that 1,4-NQ induced apoptosis at 0.1 μM in leukemic cells [30], and 9,10-PQ significantly increased DNA fragmentation at a dose of 20 μM following 12 h of exposure in A549 human pulmonary epithelial cells in vitro [31]. This study is the first, to the best of our knowledge, to demonstrate that relatively low concentrations of these quinones combined with h-PM2.5 dramatically induce cell death by apoptosis or necrosis. Furthermore, the coexistence of quinones and h-PM2.5 was observed to increase ROS generation, although the exact mechanism remains unclear.

In this study, the levels of Pb (780 ng/mg), As (150 ng/mg), Cu (180 ng/mg), Cr (91 ng/mg), Ni (98 ng/mg), and Cd (9.5 ng/mg) in h-PM2.5 were 3.12-fold, 9.4-fold, 4.4-fold, 1.5-fold, 1.8-fold, and 24.4-fold higher than those in h-ASD, respectively. It has been reported that metals with H_2_O_2_ have the ability to generate hydroxyl radicals in aqueous buffer solutions at pH 7.4 in the order Fe (II) > V (IV) > Cr (III) > Cu (I) > Co (II)> Ni (II) > Pb (II) > Cd (II) [32]. Therefore, these components in h-PM2.5 may play an important role in ROS generation due to their combination with quinones. ROS-induced oxidative stress is an important part of apoptosis [33]. Higher ROS levels can induce oxidative DNA damage and apoptosis, which contribute to allergic asthma and other respiratory diseases [34]. Enhanced formation of ROS activates cellular signaling mechanisms, inducing the inflammatory response observed in asthma and many other pulmonary conditions, such as chronic obstructive pulmonary disease, cystic fibrosis, idiopathic pulmonary fibrosis, and respiratory distress syndrome [35,36,37,38]. In addition, high ROS levels can damage the DNA, and given their roles as signaling molecules and inflammatory mediators, they can impede apoptosis and activate protooncogenes [39]. These results strongly indicate that these quinones at relatively low concentrations exert toxicity in the presence of h-PM2.5, at least in part, through increased ROS production generated by their mixture (a ROS-dependent pathway). In other words, these quinone components may be, at least in part, responsible for the toxicity of PM2.5 to respiratory cells. Further investigation is required to determine the factors responsible for the phenomenon (e.g., attaching style of quinones + PM2.5, responsible effects on Fas, tumor necrosis factor, proapoptotic proteins, etc.).

In conclusion, this study demonstrated that exposure to two types of quinones, quinones + h-PM2.5 and/or h-ASD, dose-dependently increased WST-1 reduction at lower concentrations compared to the corresponding control, whereas it markedly decreased at higher concentrations. At a lower concentration of 9,10-PQ, exposure to h-ASD or h-PM2.5 + h-ASD increased the number of late apoptotic or necrotic cells, whereas exposure to 9,10-PQ + h-PM2.5 markedly increased the number. At a higher concentration of 1,2-NQ, exposure to h-ASD or h-PM2.5 + h-ASD also increased the number of late apoptotic or necrotic cells, whereas exposure to 1,2-NQ + h-PM2.5 especially increased their number. Quinones with or without h-PM2.5, h-ASD, or h-PM2.5 + h-ASD dose-dependently increased ROS production compared to the corresponding control. In the cell-free condition, quinones with or without h-PM2.5, h-ASD, or h-PM2.5 + h-ASD also dose-dependently increased ROS production compared to the corresponding control. Specifically, quinones + h-PM2.5 significantly increased the value.

These results suggest that exposure to quinone chemicals at low concentrations combined with h-PM2.5 induces apoptosis or necrosis of bronchial epithelial cells, which is concomitant with intracellular or extracellular ROS overproduction. In contrast, h-ASD alone or combined with quinones induces apoptosis or necrosis. The different cell death patterns resulting from the coexistence of quinone chemicals may lead to different lung inflammations and injuries.

## 4. Materials and Methods

### 4.1. Cell Culture

The BEAS-2B cell line, derived from human bronchial epithelial cells transformed by an adenovirus 12-SV40 hybrid virus, was purchased from the European Collection of Cell Cultures (Salisbury, Wiltshire, UK). Airway epithelial cells were seeded in collagen-I-coated 96- or 12-well plates and incubated for 72 h to reach semiconfluence. The cells were incubated in serum-free medium LHC-9 (Life Technologies, Carlsbad, CA, USA) at 37 °C in a humidified atmosphere containing 5% CO_2_.

### 4.2. Preparation of Heated-PM2.5 and Heated-ASD

PM reference material (CRM No. 28) is atmospheric dust collected by a ventilation filter of a building in Beijing from 1996–2005 [40] and was purchased from the National Institute for Environmental Studies, Japan. The PM was classified as PM2.5 [41]. The PM2.5 was heated at 360 °C for 30 min (h-PM2.5) to exclude substances that are sensitive to heat, such as PAH [21]. We identified ten major elements in h-PM2.5, with the highest concentration of Si (228 μg/mg), followed by Ca (98 μg/mg), Al (67 μg/mg), Fe (34 μg/mg), K (17 μg/mg), Ti (3.7 μg/mg), Zn (1.9 μg/mg), Ba (1.2 μg/mg), Mn (0.99 μg/mg), and Pb (0.78 μg/mg). Additionally, minor elements were present, with the highest concentration of Cu (180 ng/mg), followed by As (150 ng/mg), Sc (100 ng/mg), Ni (98 ng/mg), V (97 ng/mg), Cr (91 ng/mg), Ce (91 ng/mg), Rb (83 ng/mg), Sb (37 ng/mg), Mo (46 ng/mg), La (44 ng/mg), Co (36 ng/mg), Se (26 ng/mg), Th (16 ng/mg), Cd (9.5 ng/mg), W (8.5 ng/mg), Cs (7.4 ng/mg), Sm (6.8 ng/mg), Hf (4.9 ng/mg), and Ta (1.1 ng/mg). The anion concentrations were SO_4_^2−^ (16 μg/mg), Cl^-^ (4.0 μg/mg), and NO_3_^−^ (0.89 μg/mg). Additionally, the concentrations of water-soluble components were SO_4_^2−^ (16 μg/mg), NO_3_^−^ (0.89 μg/mg), Ca^2+^ (78 μg/mg), Mg^2+^ (4.5 μg/mg), Na^+^ (3.5 μg/mg), and K^+^ (3.4 μg/mg). The levels of elements were analyzed by inductively coupled plasma atomic emission spectroscopy (ICP-AES, 61E Trace, and ICP-750; Thermo Jarrell-Ash, MA, USA). The ions were analyzed by an ion chromatograph (DX-100; Dionex, Sunnyvale, CA, USA). The median diameter of h-PM2.5 was 1.26 ± 0.73 μm [41]. 

ASD was purchased as a reference material (CRM No. 30, Gobi Kosa dust) from the National Institute for Environmental Studies, Japan, and was purified. To exclude substances that are sensitive to heat, such as PAH, the ASD was treated by heating at 360 °C for 30 min, resulting in heated-ASD (h-ASD) [21]. The major elemental compositions of the h-ASD were as follows: Si (280 μg/mg), Al (82 μg/mg), Ca (45 μg/mg), Fe (37 μg/mg), K (22 μg/mg), Ti (4.7 μg/mg), Zn (1.1 μg/mg), Mn (0.79 μg/mg), Ba (0.54 μg/mg), and Pb (0.25 μg/mg). The minor elemental compositions were: Sc (150 ng/mg), Rb (110 ng/mg), V (96 ng/mg), Ce (92 ng/mg), Cr (62 ng/mg), Ni (55 ng/mg), Cu (41 ng/mg), La (39 ng/mg), As (16 ng/mg), Co (16 ng/mg), Th (13 ng/mg), Cs (8.4 ng/mg), Sm (6.1 ng/mg), Hf (5 ng/mg), W (4.6 ng/mg), Mo (1.4 ng/mg), Sb (1.2 ng/mg), Ta (0.99 ng/mg), Se (<2.7 ng/mg), and Cd (<0.39 ng/mg). The concentrations of water-soluble components were SO_4_^2-^ (0.68 μg/mg), NO_3_^−^ (0.71 μg/mg), Ca^2+^ (21 μg/mg), Mg^2+^ (4.9 μg/mg), Na^+^ (2.9 μg/mg), and K^+^ (1.2 μg/mg). The median diameter of h-ASD was 2.87 ± 2.42 μm [41].

### 4.3. Experimental Protocol

After airway epithelial cells were grown to semiconfluence in LHC-9, we exposed the cells to two types of quinones (9,10-PQ [Sigma, St. Louis, MO, USA] and 1,2-NQ [Tokyo Chemical Industry Co., Ltd., Tokyo, Japan], Supplementary Figure S1) at a concentration of 0.1, 1, 10 μM, quinones + h-PM2.5 (50 μg/mL), quinones + h-ASD (50 μg/mL), and quinones + h-PM2.5 (25 μg/mL) + h-ASD (25 μg/mL) for 3 or 24 h. The exposure doses were referred to in previous studies [42]. The WST-1 reduction, cell death, and cytokine and ROS production were examined using absorptiometry, flow cytometry, and enzyme-linked immunosorbent assay (ELISA), respectively.

### 4.4. WST-1 Reduction Assay

We measured the change of tetrazolium dye (WST-1) as an external electron acceptor for reduction using the Premix WST-1 Cell Proliferation Assay System (TaKaRa Bio, Shiga, Japan). Briefly, after treatment for 21 h, the WST-1 reagent was added to each well of a 96-well plate and mixed by gentle shaking. After incubating BEAS-2B cells with the WST-1 reagent at 37 °C for 3 h, absorbance was measured at a wavelength and a reference wavelength of 450 nm and 630 nm, respectively, using an iMarkMicroplate Absorbance Reader (Bio-Rad Laboratories, Hercules, CA, USA). The results were expressed as the percentage of exposed cells relative to untreated cells (control, 0.1% DMSO).

### 4.5. Cell Death Detection via Apoptosis and Necrosis

Cell death through apoptosis and necrosis was measured using annexin V (FITC) and Ethidium Homodimer III in an Apoptotic/Necrotic/Healthy Cells Detection Kit (Promokine, Heidelberg, Germany) and a fluorescence-activated cell sorter (FACS Calibur, Becton Dickinson, San Jose, CA, USA).

Cells were exposed to quinones + h-PM2.5 (50 μg/mL), quinones + h-ASD (50 μg/mL), and quinones + h-PM2.5 (25 μg/mL) + h-ASD (25 μg/mL). After treatment for 24 h (*n* = 4), the cells were pooled together for measurement of (1) non-stain, (2) FITC-Annexin V stain, (3) Ethidium Homodimer III stain, and (4) FITC-Annexin V+ Ethidium Homodimer III stain. These (1)–(3) were used to determine autofluorescence and AnnexinV and Ethidium homodimer positive areas for each group, and each gating area (Q1, necrotic cells; Q2, late apoptotic or necrotic cells; Q3, early apoptotic cells; Q4, healthy cells) was defined. 

### 4.6. Quantitation of Inflammatory Proteins in the Culture Supernatant

After cell treatment for 24 h, the cell culture medium was collected and centrifuged at 300× *g* for 5 min to remove floating cells. The resulting supernatant was stored at −80 °C until further use. The levels of interleukin (IL)-6 and IL-8 in the culture medium were quantified using an ELISA kit (Thermo Scientific, Waltham, MA, USA), following the manufacturer’s instructions. The absorbance was measured on the iMark microplate absorbance reader at 450 nm with a reference wavelength of 550 nm.

### 4.7. Quantification of ROS Generation

To measure intracellular and cell-free ROS production, a fluorescent probe, 5-(and-6)-chloromethyl-2′,7′-dichlorodihydrofluorescein diacetate acetyl ester (CM-H_2_DCFDA), was used. For intracellular ROS measurements, cells were incubated with 5 μM CM-H_2_DCFDA for 30 min to allow the dye to enter the cells. The cells were then washed to remove the extracellular dye, and the cells were exposed to two types of quinones (9,10-PQ and 1,2-NQ) at a concentration of 0.1, 1, and 10 μM: quinones + h-PM2.5 (50 μg/mL), quinones + h-ASD (50 μg/mL), and quinones + h-PM2.5 (25 μg/mL) + h-ASD (25 μg/mL). Fluorescence intensity during 0–3 h was measured (excitation and emission at 485 nm and 530 nm, respectively).

To measure cell-free ROS, 1 mM CM-H_2_DCFDA was hydrolyzed with 0.01 M NaOH at 37 °C under dark conditions for 30 min to react with ROS under cell-free conditions. We mixed two types of quinones (9,10-PQ and 1,2-NQ) at concentrations of 0.1, 1, and 10 μM with quinones + h-PM2.5 (50 μg/mL), quinones + h-ASD (50 μg/mL), and quinones + h-PM2.5 (25 μg/mL) + h-ASD (25 μg/mL). We then added 5 μM CM-H_2_DCFDA, and fluorescence intensity was measured for 3 h using excitation and emission wavelengths of 485 nm and 530 nm, respectively.

### 4.8. Statistical Analysis

The data were presented as the mean ± standard deviation for each experimental group (*n* = 4). The significance of variation among different groups was determined by a one-way analysis of variance. Differences among groups were analyzed using Tukey’s multiple comparison tests (Excel Statistics, Social Survey Research Information Co., Ltd., Tokyo, Japan). A *p*-value < 0.05 was considered statistically significant. For the detection of cell death via apoptosis and necrosis, the cells were pooled together after treatment for 24 h (*n* = 4).

## Data Availability

The data presented in this study are available upon request from the corresponding author.

## Supplementary Materials

The following supporting information can be downloaded at: https://www.mdpi.com/article/10.3390/ijms241310544/s1.

## References

[1] Alghamdi M.A., Shamy M., Redal M.A., Khoder M., Awad A.H., Elserougy S. (2014). Microorganisms associated particulate matter: A preliminary study. Sci. Total. Environ..

[2] Cheng Z., Luo L., Wang S., Wang Y., Sharma S., Shimadera H., Wang X., Bressi M., de Miranda R.M., Jiang J. (2016). Status and characteristics of ambient PM_2.5_ pollution in global megacities. Environ. Int..

[3] Guastadisegni C., Kelly F.J., Cassee F.R., Gerlofs-Nijland M.E., Janssen N.A., Pozzi R., Brunekreef B., Sandström T., Mudway I. (2010). Determinants of the Proinflammatory Action of Ambient Particulate Matter in Immortalized Murine Macrophages. Environ. Health Perspect..

[4] Wang Y., Zu Y., Huang L., Zhang H., Wang C., Hu J. (2018). Associations between daily outpatient visits for respiratory diseases and ambient fine particulate matter and ozone levels in Shanghai, China. Environ. Pollut..

[5] Zhou M., He G., Liu Y., Yin P., Li Y., Kan H., Fan M., Xue A., Fan M. (2015). The associations between ambient air pollution and adult respiratory mortality in 32 major Chinese cities, 2006–2010. Environ. Res..

[6] Lyu Y., Zhou J., Li J., Li J., Hu G., Wang L., Wang L., Han J., Wang D. (2022). Alterations of IL-1beta and TNF-alpha expression in RAW264.7 cell damage induced by two samples of PM_2.5_ with different compositions. Sci. Prog..

[7] Sumi D., Kumagai Y. (2007). Chemical biology of 1,2-naphthoquinone, a novel air pollutant that affects signal transduction pathways. YAKUGAKU ZASSHI.

[8] Bolton J.L., Dunlap T. (2017). Formation and Biological Targets of Quinones: Cytotoxic versus Cytoprotective Effects. Chem. Res. Toxicol..

[9] Kepley C.L., Lauer F.T., Oliver J.M., Burchiel S.W. (2003). Environmental polycyclic aromatic hydrocarbons, benzo(a) pyrene (BaP) and BaP-quinones, enhance IgE-mediated histamine release and IL-4 production in human basophils. Clin. Immunol..

[10] Valderrama J.A., Leiva H., Rodríguez J.A., Theoduloz C., Schmeda-Hirshmann G. (2008). Studies on quinones. Part 43: Synthesis and cytotoxic evaluation of polyoxyethylene-containing 1,4-naphthoquinones. Bioorg. Med. Chem..

[11] Shinyashiki M., Eiguren-Fernandez A., Schmitz D.A., Di Stefano E., Li N., Linak W.P., Cho S.-H., Froines J.R., Cho A.K. (2009). Electrophilic and redox properties of diesel exhaust particles. Environ. Res..

[12] Hiyoshi K., Takano H., Inoue K.-I., Ichinose T., Yanagisawa R., Tomura S., Cho A.K., Froines J.R., Kumagai Y. (2005). Effects of a single intratracheal administration of phenanthraquinone on murine lung. J. Appl. Toxicol..

[13] Inoue K., Takano H., Hiyoshi K., Ichinose T., Sadakane K., Yanagisawa R., Tomura S., Kumagai Y. (2007). Naphthoquinone enhances antigen-related airway inflammation in mice. Eur. Respir. J..

[14] Inoue K.-I., Takano H., Ichinose T., Tomura S., Yanagisawa R., Sakurai M., Sumi D., Cho A.K., Hiyoshi K., Kumagai Y. (2007). Effects of naphthoquinone on airway responsiveness in the presence or absence of antigen in mice. Arch. Toxicol..

[15] Mueller A., Ulrich N., Hollmann J., Sanchez C.E.Z., Rolle-Kampczyk U.E., von Bergen M. (2019). Characterization of a multianalyte GC-MS/MS procedure for detecting and quantifying polycyclic aromatic hydrocarbons (PAHs) and PAH derivatives from air particulate matter for an improved risk assessment. Environ. Pollut..

[16] Connellan S.J. (2017). Lung diseases associated with hydrocarbon exposure. Respir. Med..

[17] Xu Y., Li Z., Liu Y., Pan B., Peng R., Shao W., Yang W., Chen M., Kan H., Ying Z. (2021). Differential Roles of Water-Insoluble and Water-Soluble Fractions of Diesel Exhaust Particles in the Development of Adverse Health Effects Due to Chronic Instillation of Diesel Exhaust Particles. Chem. Res. Toxicol..

[18] Sadakane K., Ichinose T., Takano H., Yanagisawa R., Inoue K.-I., Kawazato H., Yasuda A., Hayakawa K. (2013). Organic Chemicals in Diesel Exhaust Particles Enhance Picryl Chloride-Induced Atopic Dermatitis in NC/Nga Mice. Int. Arch. Allergy Immunol..

[19] Inoue K.-I., Takano H., Yanagisawa R., Sakurai M., Abe S., Yoshino S., Yamaki K., Yoshikawa T. (2007). Effects of Components Derived from Diesel Exhaust Particles on Lung Physiology Related to Antigen. Immunopharmacol. Immunotoxicol..

[20] Yanagisawa R., Takano H., Inoue K.-I., Ichinose T., Sadakane K., Yoshino S., Yamaki K., Yoshikawa T., Hayakawa K. (2006). Components of diesel exhaust particles differentially affect Th1/Th2 response in a murine model of allergic airway inflammation. Clin. Exp. Allergy J. Br. Soc. Allergy Clin. Immunol..

[21] He M., Ichinose T., Ito T., Toriba A., Yoshida S., Kaori S., Nishikawa M., Sun G., Shibamoto T. (2019). Investigation of inflammation inducing substances in PM2.5 particles by an elimination method using thermal decomposition. Environ. Toxicol..

[22] Rodríguez-Cotto R.I., Ortiz-Martínez M.G., Jiménez-Vélez B.D. (2015). Organic extracts from African dust storms stimulate oxidative stress and induce inflammatory responses in human lung cells through Nrf2 but not NF-κB. Environ. Toxicol. Pharmacol..

[23] Honda A., Chowdhury P.H., Ito S., Okano H., Onishi T., Kawaryu Y., Ueda K., Takano H. (2017). Synergic effects of 9,10-phenanthrenequinone and cadmium on pro-inflammatory responses in airway epithelial cells. Environ. Toxicol. Pharmacol..

[24] Goler A.M.Y., Jannuzzi A.T., Bayrak N., Yıldız M., Yıldırım H., Otsuka M., Fujita M., Radwan M.O., TuYuN A.F. (2022). In Vitro and In Silico Study to Assess Toxic Mechanisms of Hybrid Molecules of Quinone-Benzocaine as Plastoquinone Analogues in Breast Cancer Cells. ACS Omega.

[25] Menchinskaya E., Chingizova E., Pislyagin E., Likhatskaya G., Sabutski Y., Pelageev D., Polonik S., Aminin D. (2021). Neuroprotective Effect of 1,4-Naphthoquinones in an *In Vitro* Model of Paraquat and 6-OHDA-Induced Neurotoxicity. Int. J. Mol. Sci..

[26] Tan A.S., Berridge M.V. (2010). Evidence for NAD(P)H:quinone oxidoreductase 1 (NQO1)-mediated quinone-dependent redox cycling via plasma membrane electron transport: A sensitive cellular assay for NQO1. Free. Radic. Biol. Med..

[27] Chang J., Go Y.Y., Park M.K., Chae S.-W., Lee S.-H., Song J.-J. (2016). Asian Sand Dust Enhances the Inflammatory Response and Mucin Gene Expression in the Middle Ear. Clin. Exp. Otorhinolaryngol..

[28] Yadav M.K., Go Y.Y., Chae S.-W., Park M.K., Song J.-J. (2020). Asian Sand Dust Particles Increased Pneumococcal Biofilm Formation in vitro and Colonization in Human Middle Ear Epithelial Cells and Rat Middle Ear Mucosa. Front. Genet..

[29] Piao M.J., Ahn M.J., Kang K.A., Ryu Y.S., Hyun Y.J., Shilnikova K., Zhen A.X., Jeong J.W., Choi Y.H., Kang H.K. (2018). Particulate matter 2.5 damages skin cells by inducing oxidative stress, subcellular organelle dysfunction, and apoptosis. Arch. Toxicol..

[30] Portilho A.J.D.S., da Silva E.L., Bezerra E.C.A., Gomes C.B.D.S.M.R., Ferreira V., de Moraes M.E.A., da Rocha D.R., Burbano R.M.R., Moreira-Nunes C.A., Montenegro R.C. (2022). 1,4-Naphthoquinone (CNN1) Induces Apoptosis through DNA Damage and Promotes Upregulation of *H2AFX* in Leukemia Multidrug Resistant Cell Line. Int. J. Mol. Sci..

[31] Sugimoto R., Kumagai Y., Nakai Y., Ishii T. (2005). 9,10-Phenanthraquinone in diesel exhaust particles downregulates Cu,Zn–SOD and HO-1 in human pulmonary epithelial cells: Intracellular iron scavenger 1,10-phenanthroline affords protection against apoptosis. Free. Radic. Biol. Med..

[32] Valavanidis A., Vlahoyianni T., Fiotakis K. (2005). Comparative study of the formation of oxidative damage marker 8-hydroxy-2′-deoxyguanosine (8-OHdG) adduct from the nucleoside 2′-deoxyguanosine by transition metals and suspensions of particulate matter in relation to metal content and redox reactivity. Free. Radic. Res..

[33] Belhadj Slimen I., Najar T., Ghram A., Dabbebi H., Ben Mrad M., Abdrabbah M. (2014). Reactive oxygen species, heat stress and oxidative-induced mitochondrial damage. A review. Int. J. Hyperth..

[34] Jiang L., Diaz P.T., Best T.M., Stimpfl J.N., He F., Zuo L. (2014). Molecular characterization of redox mechanisms in allergic asthma. Ann. Allergy Asthma Immunol..

[35] Dailah H.G. (2022). Therapeutic Potential of Small Molecules Targeting Oxidative Stress in the Treatment of Chronic Obstructive Pulmonary Disease (COPD): A Comprehensive Review. Molecules.

[36] Moliteo E., Sciacca M., Palmeri A., Papale M., Manti S., Parisi G.F., Leonardi S. (2022). Cystic Fibrosis and Oxidative Stress: The Role of CFTR. Molecules.

[37] Estornut C., Milara J., Bayarri M.A., Belhadj N., Cortijo J. (2022). Targeting Oxidative Stress as a Therapeutic Approach for Idiopathic Pulmonary Fibrosis. Front. Pharmacol..

[38] von Knethen A., Heinicke U., Laux V., Parnham M.J., Steinbicker A.U., Zacharowski K. (2022). Antioxidants as Therapeutic Agents in Acute Respiratory Distress Syndrome (ARDS) Treatment—From Mice to Men. Biomedicines.

[39] Vuong H.G., Nguyen T.Q., Nguyen H.C., Nguyen P.T., Ho A.T.N., Hassell L. (2020). Efficacy and Safety of Crizotinib in the Treatment of Advanced Non-Small-Cell Lung Cancer with ROS1 Rearrangement or MET Alteration: A Systematic Review and Meta-Analysis. Target. Oncol..

[40] Mori I., Sun Z., Ukachi M., Nagano K., McLeod C.W., Cox A.G., Nishikawa M. (2008). Development and certification of the new NIES CRM 28: Urban aerosols for the determination of multielements. Anal. Bioanal. Chem..

[41] He M., Ichinose T., Yoshida S., Nishikawa M., Sun G., Shibamoto T. (2019). Role of iron and oxidative stress in the exacerbation of allergic inflammation in murine lungs caused by urban particulate matter <2.5 μm and desert dust. J. Appl. Toxicol..

[42] Chowdhury P.H., Kitamura G., Honda A., Sawahara T., Hayashi T., Fukushima W., Kudo H., Ito S., Yoshida S., Ichinose T. (2017). Synergistic effect of carbon nuclei and polyaromatic hydrocarbons on respiratory and immune responses. Environ. Toxicol..

