# Supplementary material for: Differential Pattern of Cell Death and ROS Production in Human Airway Epithelial Cells Exposed to Quinones Combined with Heated-PM2.5 and/or Asian Sand Dust
Source: Int J Mol Sci. 2023 Jun 23;24(13):10544. doi: 10.3390/ijms241310544 (PMC10341977; doi:10.3390/ijms241310544)
Supplement: Supplementary file 1 [file ijms-24-10544-s001.zip › ijms-2438195-supplementary.pdf]

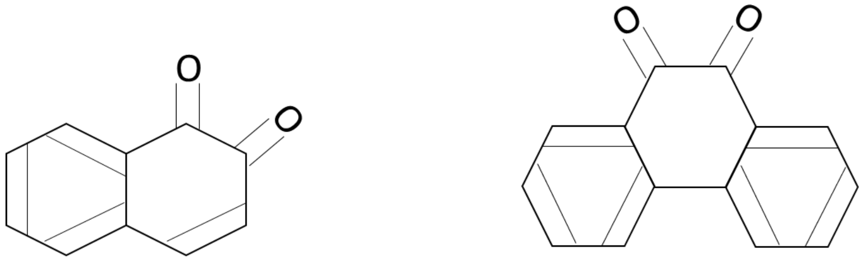

1,2-NQ **9,10-PQ**

Figure S1. Chemical structural formula of 1, 2-NQ and 9, 10-PQ.

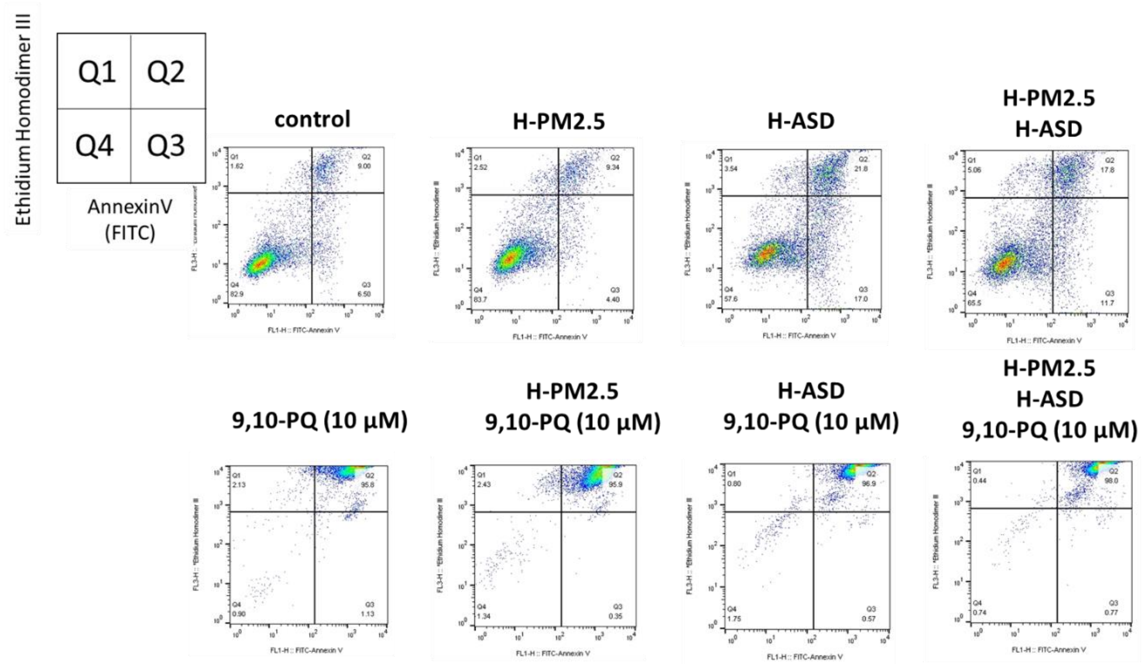

Figure S2. Combined effect of 9, 10-PQ (10 ◎M) and h-PM2.5 and/or h-ASD on apoptosis and necrosis of airway epithelial cells. Dot-plot of flow cytometry, Q1, necrosis cell; Q2, late apoptotic or necrotic cells; Q3, early apoptosis cell; Q4, healthy cell.
